# Anatomically compliant modes of variations: New tools for brain connectivity

**DOI:** 10.1371/journal.pone.0292450

**Published:** 2023-11-07

**Authors:** Letizia Clementi, Eleonora Arnone, Marco D. Santambrogio, Silvana Franceschetti, Ferruccio Panzica, Laura M. Sangalli

**Affiliations:** 1 MOX - Department of Mathematics, Politecnico di Milano, Milan, Italy; 2 Department of Electronics, Information and Bioengineering, Politecnico di Milano, Milan, Italy; 3 CHDS, Center for Health Data Science, Human Technopole, Milan, Italy; 4 Department of Management, University of Turin, Turin, Italy; 5 Fondazione IRCCS Istituto Neurologico “C. Besta”, Milan, Italy; IRCCS San Raffaele Scientific Research Institute, ITALY

## Abstract

Anatomical complexity and data dimensionality present major issues when analysing brain connectivity data. The functional and anatomical aspects of the connections taking place in the brain are in fact equally relevant and strongly intertwined. However, due to theoretical challenges and computational issues, their relationship is often overlooked in neuroscience and clinical research. In this work, we propose to tackle this problem through Smooth Functional Principal Component Analysis, which enables to perform dimensional reduction and exploration of the variability in functional connectivity maps, complying with the formidably complicated anatomy of the grey matter volume. In particular, we analyse a population that includes controls and subjects affected by schizophrenia, starting from fMRI data acquired at rest and during a task-switching paradigm. For both sessions, we first identify the common modes of variation in the entire population. We hence explore whether the subjects’ expressions along these common modes of variation differ between controls and pathological subjects. In each session, we find principal components that are significantly differently expressed in the healthy vs pathological subjects (with p-values < 0.001), highlighting clearly interpretable differences in the connectivity in the two subpopulations. For instance, the second and third principal components for the rest session capture the imbalance between the Default Mode and Executive Networks characterizing schizophrenia patients.

## Introduction

Functional Magnetic Resonance Imaging (fMRI) based studies are standard in neuroscience to explore functional connections in the brain and how they can be affected by psychiatric and neurological diseases [1]. Neurodegenerative pathologies, indeed, affect both anatomical and functional connections in the brain, resulting also in modifications in their relationship (see, e.g., [2, 3]). Nonetheless, the analysis of this type of data still presents relevant theoretical and technical challenges, due for instance to the data dimensionality and to the complicated anatomy of the brain.

The high dimensionality of fMRI data poses major statistical and computational issues, yet to be fully addressed [4–6]. Various alternative approaches to dimensionality reduction of fMRI data have been proposed in the literature, including adaptations of Principal Component Analysis (PCA) to the context of large-scale datasets (see, e.g., [7]). Moreover, Blood-Oxygenation Level Dependent (BOLD) signals have also been considered as functional data [8–11], and analysed through functional PCA methods (see, e.g., [12, 13]). However, high dimensionality is not the only challenge when applying PCA to the analysis of fMRI data.

Other critical issues are posed for instance by anatomical complexity. Indeed, the brain cortex has a highly complicated anatomy, characterized by the presence of gyri and sulci, fundamental to its correct functioning. Of course, fMRI signals observed within the same brain area are usually related. It should though be noted that areas carrying out distinct functions may be close in three-dimensional space, due to the aforementioned highly-folded structure of the brain. Because of these aspects, the application of classical multivariate dimensional reduction techniques, as well as of state-of-the arts methods for functional data, such as functional PCA, may lead to unsatisfactory or misleading results. The former techniques may present unsatisfactory results, since they disregard the spatial structure in the data, and are hence incapable of borrowing information from signals observed at nearby locations, where they are correlated. The latter techniques, based on functional data analysis, may instead produce artifacts, since these methods rely on the Euclidean metrics, and hence neglect the highly non-Euclidean brain anatomy, and its effects on the BOLD signals. See also the discussions in [14–16].

For these reasons, [15] proposed a form of PCA able to handle observations located over a complicated two-dimensional manifold, and applied it to Functional Connectivity (FC) maps computed over the cortical surface of the brain. In particular, they consider a penalization term that permits to include the manifold geometry into the estimation problem, thus appropriately accounting for the complicated shape of the cerebral cortex [17]. The extracted eigenfunctions may hence be treated with classical Functional Data Analysis tools. Others advanced data analysis methods capable of complying with the complex anatomy of the cortex have been proposed in, e.g., [14, 18–20].

In this work, we aim to expand the approach proposed by [15] to data observed within volumetric domains with complicated shapes, considering FC maps computed within the grey matter volume. We propose a method to compute principal components of connectivity maps, where we appropriately consider domain-specific distances, within the grey matter volume, respecting the highly non-Euclidean anatomy of the brain. In particular, the method features a regularizing term, defined over the grey matter volume, that leads to the computation of distances within this volume, and not across sulci or other concavities in the grey matter. Moreover, to address the computational issues linked to the dimensionality of data, the considered technique leverages an appropriate finite element discretization, defined over the tetrahedral mesh representing the grey matter volume, over which the fMRI data are mapped. By merging approaches and ideas from statistics and from scientific computing, the considered Smooth Functional Principal Component Analysis (SF-PCA) tackles the challenges posed by both anatomical complexity and data dimensionality, permitting to explore the variability in neuroimaging signals associated with neural connectivity in the grey matter, complying with its complicated structure, at a sustainable computational cost.

To prove the validity of SF-PCA in this context, we analyze data from an experimental population composed of subjects affected by schizophrenia and by controls. Schizophrenia is a severe psychiatric condition characterized by hallucinations, delusions, and impaired cognitive function, hypothesized to result from abnormal anatomical neural connectivity, and a consequent decoupling of the brain’s integrative thought processes [21–24]. The impact of this pathology on brain connectivity has been widely explored, both through resting-state and task-based fMRI experiments, leading to a well-defined description of the impact of schizophrenia on brain connections [25–27]. For this purpose, we consider the FC maps computed on fMRI data acquired during resting-state and also while carrying out a task based on the switching paradigm. We assess the adequacy of the proposed SF-PCA method to analyze FC maps computed on grey matter volume domains. In particular, we first consider the entire population, including both controls and subjects affected by schizophrenia, and we extract the main common modes of variation of brain connectivity, identified by the Smooth Functional Principal Components (SF-PCs) of the FC maps. We then explore whether the corresponding SF-PCs scores, that describe the subjects’ expression along these common modes of variation, differ significantly between the healthy and pathological subgroups. A significant difference in the expression of the main modes of variation would indeed highlight differences in the expression of the associated connectivity patterns. This aspect is further explored by Linear Discriminant Analysis of the SF-PCs scores, considering the Area Under the Curve of its receiver operating characteristic curve [28]. A comparison with standard MV-PCA highlights the advantages of the proposed method in terms of interpretability of the obtained principal components. Finally, we discuss how the differences in the expression of the SF-PCs corroborate previous findings in literature and also shed new light on connectivity impairment in schizophrenia.

## Materials and methods

This section describes the fMRI dataset employed in the present study, the experimental protocol, and the population enrolled, alongside the preprocessing and analysis pipelines.

### Data

The data analyzed in this work has been originally collected and made available by [29]. Participants were reached through a patient-oriented strategy involving local clinics and online portals, different from the methods employed to recruit healthy volunteers, who were enlisted by community advertisements from the Los Angeles area. After receiving a verbal explanation of the study, participants gave written informed consent following procedures approved by the Institutional Review Boards at UCLA and the Los Angeles County Department of Mental Health [29]. All data was collected before 2016 (the date on which the acquisition started is undisclosed) and first published on 27 January 2016. In this work we are employing the preprocessed version of the dataset by [30], made available by the Consortium for Neuropsychiatric Phenomics at UCLA. The last update of the preprocessed version of the dataset is dated 21st April 2020. In none of the stages of this work, we had access to information that could identify the participants in the study. The fMRI data were acquired through a 3T Siemens Trio scanner. T1-weighted high-resolution anatomical scans were collected with the following parameters: slice thickness = 1mm for 176 slices, TR = 1.9s, TE = 2.26ms, matrix = 256x256, FOV = 250mm. The T2*-weighted BOLD fMRI sequence parameters instead are: slice thickness = 4mm for 34 slices, TR = 2s, TE = 30ms, flip angle = 90°, matrix = 64×64, FOV = 192mm. For both the considered sessions, the authors acquired 34 volumes. The data are made available as coregistered to the skull-stripped ICBM 152 Nonlinear Asymmetrical template version 2009c [31, 32]. The registration to the template allows to indirectly account for structural dissimilarities characterizing the two groups, among which the atrophy affecting schizophrenia patients.

The complete preprocessing and alignment pipeline, firstly proposed and standardized in [33], is described in [30]. Nonetheless, for better clarity, the main steps performed can be summarized in three main blocks:

- **T1w preprocessing**: bias field is addressed via ANTs N4BiasFieldCorrection v2.1.08 [34], skullstripping using antsBrainExtraction.sh v2.1.0, and coregistration to the ICBM 152 Nonlinear Asymmetrical template version 2009c9 [31, 32] through ANTs v2.1.0 [35].
- **Surface estimation** from the bias field corrected T1w, through antsBrainExtraction. The skull-stripping is then performed via FreeSurfer v6.0.0 [36].
- **EPI preprocessing**: motion artifacts and geometric distortions are removed through MCFLIRT v5.0.9 [37]. Skull-stripping is perfomed via BET and 3d AutoMask, finally coregistration to T1w is applied via FreeSurfer. The EPI preprocessing is performed in a single step using antsApplyTransformations v2.1.0, a tool performing a non-linear registration step addressing motion correcting, transformation to T1 weighted space and MNI template warp.

### Participants

The dataset contains the fMRI scans of patients affected by either schizophrenia, bipolar disorder, or ADHD plus the control group. The fMRI were acquired during rest and while performing different tasks. Exclusion criteria were: left-handedness, pregnancy, history of head injury with loss of consciousness, diagnoses ascribable to more than one patient group or contraindications to undergo fMRI scanning.

For the present study, we select a subsample of the dataset, referring to 120 subjects from the control (CTRL) group, and 50 affected by schizophrenia (SCHZ). We analyze their resting state (REST) fMRI. Among the available task-based fMRIs, we consider those referring to the task-switching (SWITCH) paradigm. A control subject did not perform the switching task, thus the resulting population comprises 120 CTRL fMRIs for the REST session and 119 fMRIs for the SWITCH one. Instead, all the 50 SCHZ subjects participated in both sessions. Median age is 28 years (interquartile range: 15 years) for CTRL and 37 years (interquartile range: 14 years) for SCHZ. The percentage of males is 51.26% for CTRL and 76.00% for SCHZ.

### Experimental paradigm

The experimental paradigm consists of two separate sessions. During each session, the fMRI is acquired while the subject performs a task. All the procedures were approved by the Institutional Review Boards at UCLA and the Los Angeles County Department of Mental Health. In the present work, as above mentioned, we consider two fMRI sessions, acquired during:

- REST: the participants were asked to relax and keep their eyes open. They were not presented any stimuli or asked to respond during the scan. This session lasted 304 seconds.
- SWITCH: the participants were presented an image plus an instruction cue describing how to react to the image. Each image included a shape and a colour, from a set of four (either a red triangle, a red circle, a green triangle, or a green circle). Participants were given instruction to respond either to the stimuli’s color (possible cues: “COLOR” or “C”) or shape (possible cues: “SHAPE” or “S”). On 33% of trials, the instructions switched, such that participants were instructed to switch from responding from shape to color, or vice versa. On the remaining 67% of trials, the instructions remained the same, but the cues changed (e.g. from “SHAPE” to “S”). This task was designed to measure the changes in reaction time between trials requiring versus not requiring a switch in responding.

### Analysis

This subsection describes in detail the analysis pipeline, providing also indications on the Matlab [38] and R [39] software used.

#### Brain mesh creation and data preprocessing

In order to apply Smooth Functional Principal Component Analysis (SF-PCA) we need a Finite Element mesh that discretizes the common brain volume, given by the ICBM 152 Nonlinear Asymmetrical template (version 2009c) [31, 32], to which all subjects’ data have been aligned, as detailed in the preprocessing steps above. With some abuse of notation, we denote by D both the original template domain, as well as the tetrahedral mesh discretizing it. The mesh D is computed through the Brain2Mesh Matlab toolbox (version 0.80) [40, 41]. The number of mesh nodes is automatically computed by the Brain2Mesh tool, with an algorithm that considers the tissues to be segmented and the desired mesh density. In this work, we selected the finest mesh possible with respect to the data resolution. Specifically, approximately 46 000 voxels of the original fMRI contain information regarding brain activity, including both the grey and the white matter. Since we only consider the grey matter, this leads to a mesh of 36 035 nodes. Each mesh node is associated with a cerebral region according to the Hammers’ atlas (rigidly registered to the template) [42, 43] and to a voxel of the fMRI. Thus, each node in the grey matter mesh corresponds to a BOLD signal, that we highpass filter at 0.01 Hz to remove the linear drift and low-frequency noise [1].

#### Connectivity map

For each subject, we compute the Functional Connectivity (FC) maps through a seed-based technique, a straightforward approach to FC computation [1]. Previous research has shown the effectiveness of this method, also in comparison to more sophisticated approaches (see, e.g., [44, 45]). The seeds, named regions of interest, are selected regions which, according to previous findings, are of particular interest with respect to the considered task. In particular, we consider the following seeds:

- Seed for REST session: we consider as the region of interest the anterior part of the cingulate gyrus, in the left hemisphere, that corresponds to the label number 24 of the Hammer’s atlas [27]. In the considered brain discretization, this seed region is discretized in *m*_*seed*_ = 113 nodes. Previous studies observed anatomical abnormalities in the grey matter of patients affected by schizophrenia [46] in association with a loss of connectivity in this region [47, 48]. Moreover, this region is hypothesized to play a pivotal role in the onset and progression of the pathology [49].
- Seed for SWITCH session: we consider as the region of interest the middle frontal gyrus of the right hemisphere, corresponding to the Hammer’s atlas label number 29. This seed region is discretized in *m*_*seed*_ = 1007 nodes. The strength of functional connections between the right and left middle frontal gyrus has been found to predict performances in task-switching in subjects affected by cognitive impairment [50, 51].

For each subject, we compute the FC maps through the following preprocessing steps:

1. we associate to each node **p**_*j*_ (for *j* = 1, …, *m* and *m* = 36 035), of the grey matter volume D, its BOLD signal **t**_*j*_;
2. we compute the mean BOLD signal $\bar{t}$ over the seed region;
3. we associate to each node **p**_*j*_ a single value of correlation *ρ*_*j*_, that corresponds to the Pearson correlation coefficient between the mean time series over the seed region $\bar{t}$ and the BOLD **t**_*j*_ in **p**_*j*_;
4. we compute the z-Fisher’s transform of the Pearson’s correlation in each node, in order to stabilize variance [1].

Finally, for both the REST and SWITCH, we organize the extracted z-scores in a matrix **Z** of size *n* × *m*, where *n* is the number of subjects enrolled in the study and *m* the number of nodes in the mesh. As indicated above, the number of subjects *n* is 170 for REST and 169 for SWITCH. The number of mesh nodes *m* is 36 035 for each session, as it does not depend on the considered seed.

#### Smooth functional principal component analysis

In our data setting, each row of the data matrix **Z** is the noisy evaluation of the connectivity map for one subject, defined over the grey matter volume D, and evaluated at the *m* nodes **p**_*j*_ of its tetrahedral mesh discretization. Our goal is to extract some components that describe the strongest modes of variations in the computed connectivity maps, that is in the data matrix **Z**, similarly to the standard MultiVariate Principal Component Analysis (MV-PCA). However, differently from MV-PCA, where the components are vectors in $R^{m}$, we would like these components to be functions *f* defined over the whole domain D, i.e., f:D→R, and to be smooth. This would permit to appropriately consider the spatial structure in the observed map. To do so, we embed our analysis in the framework of Functional Data Analysis [8–11]. In particular, we consider the Smooth Functional Principal Component Analysis (SF-PCA) method, previously proposed by [15] for the analysis of functional maps referring to the cortical surface, and we extend it to the case of data referred to the grey matter.

The proposed SF-PCA approach relies on the computation of the principal components as a low-rank approximation of the data matrix [52], and proceeds to estimate the components sequentially, starting from the first one. Specifically, for each component, we consider a rank-1 approximation to a PCA problem for zero-centered random variables. With abuse of notation, we continue to denote by **Z** the zero-centered data matrix. The rank-1 approximation problem involves the minimization of the functional

$$\begin{array}{c} \sum_{j=1}^{m}\sum_{i=1}^{n}{\left\{z_{ji}-s_{i}f\left(p_{j}\right)\right\}}^{2} \end{array}$$

where *z*_*ji*_ is the value of the zero-centered connectivity map, evaluated at the mesh node **p**_*j*_ for the *i*-th subject, *f* is the component we aim to estimate, and **s** = (*s*_1_, …, *s*_*n*_)^⊤^ is the associated score vector. The above functional does not account for the desired smoothness of the PC *f*. Therefore, following a common approach in regularized regression, we add to the functional the term $s^{⊤}s\int_{D}{\left(\Delta f\right)}^{2}$, which penalizes functions with high oscillations. Specifically, the operator Δ*f* is the Laplacian of *f*, defined as

$$\Delta f=\frac{\partial^{2}f}{\partial p_{x}^{2}}+\frac{\partial^{2}f}{\partial p_{y}^{2}}+\frac{\partial^{2}f}{\partial p_{z}^{2}}$$

and measures the local curvature of the function *f*, as defined on the domain D. Therefore, the first principal component $\hat{f}$ and the first score vector $\hat{s}$ are computed minimizing the functional:

$$\begin{array}{c} \sum_{j=1}^{m}\sum_{i=1}^{n}{\left\{z_{ji}-s_{i}f\left(p_{j}\right)\right\}}^{2}+\lambda s^{⊤}s\int_{D}{\left(\Delta f\right)}^{2} \end{array}$$ (1)

where λ > 0 is a tuning parameter, usually referred to as smoothing parameter, that regulates the relative magnitude of the two terms in the functional. In particular, large values of λ produce a very smooth functional component, while small values of λ return a noisier principal component, closer to the multivariate one. It should be noticed that the inclusion in the estimation functional of the regularizing term $s^{⊤}s\int_{D}{\left(\Delta f\right)}^{2}$, that is defined over the spatial domain D, makes the estimation problem domain-specific. Indeed, to return a smooth PC *f*, the method borrows information from data observed at nearby locations, i.e., data *z*_*ji*_ observed at spatial locations **p**_*j*_ that are close in the grey matter volume D, where the distance is naturally computed within the domain D. Because of this, the method returns estimated PC functions that comply with the complex brain shape and do not artificially smooth data at locations that are close in 3D space, but far away in the grey matter volume, as they are for instance separated by a sulcus.

The functional in Eq (1) is written in a continuous form for *f* on the grey matter volume. However, to carry out the minimization problem, such a problem is discretized with linear finite elements. In particular, we represent the PC function *f* by a linear finite element function, defined on the grey matter mesh. A linear finite element function is defined by the values that it assumes at the mesh nodes and it is linear over each mesh element, i.e., over each tetrahedron. Therefore, each linear finite element function is uniquely associated with a vector of dimension *m*, the number of mesh nodes. The discretization of the integral of the square of the Laplacian of *f*, in the second term of the functional (1), is computed following a mixed finite element approach, similarly to the manifold case in [15]. Moreover, the minimization problem is solved in a 2-step iterative algorithm: in the first step *f* is kept fixed and the optimization is performed in **s**, while in the second step **s** is kept fix and the optimization is performed in *f*. As initial value for *f*, the first multivariate PC is used. Finally, as mentioned above, the subsequent components are computed with the same 2-step iterative algorithm, after subtracting the previous principal components from the data matrix **Z**.

We apply the described SF-PCA separately to the two sessions, REST and SWITCH, setting λ = 10^−1^. Alongside, we extract the corresponding PC scores, that indicate the subjects’ expressions on the common modes of variation. The method is implemented through the R/C++ library fdaPDE [53].

#### Group comparison and SF-PCs interpretation

For each session, we carry out SF-PCA on the entire population, including healthy and pathological traces, to identify the common modes of variation. Once the main modes of variation, in the entire population, are identified, we look for differences in their expression between the two subgroups of healthy and pathological subjects. Indeed, a significant difference in the expression of the main modes of variation, i.e., on the PC scores, can highlight differences in the expressions of the associated connectivity patterns. To do so, we first compare the SF-PCs’ scores distribution of the SCHZ and CTRL groups through a Wilcoxon test. We set the significance threshold at *α* = 0.01. We further explore differences in the SF-PC scores through an Linear Discriminant Analysis (LDA) in a leave-one-out cross-validation; the metric employed for evaluation is the Area Under the Curve (AUC) of its receiving operating curve [28]. For a comparison with previous approaches present in literature, we replicate such analysis with the standard multivariate PCA (MV-PCA).

We finally evaluate if the diagnosis-based differences highlighted by SF-PCA can provide meaningful insights into the pathology in analysis. To do so, we investigate if the modes of variation highlighted by the SF-PCs comply with previous findings in the state of the art.

Given the unbalance, in terms of sex and age, of the SCHZ and CTRL groups, we also evaluate if the emerged differences can be linked to such covariates. For this purpose, we compare the SF-PCs’ scores distributions for the two sexes through Wilcoxon tests, and the Pearson correlation between the SF-PCs’ scores and the age.

## Results

**Fig 1:**
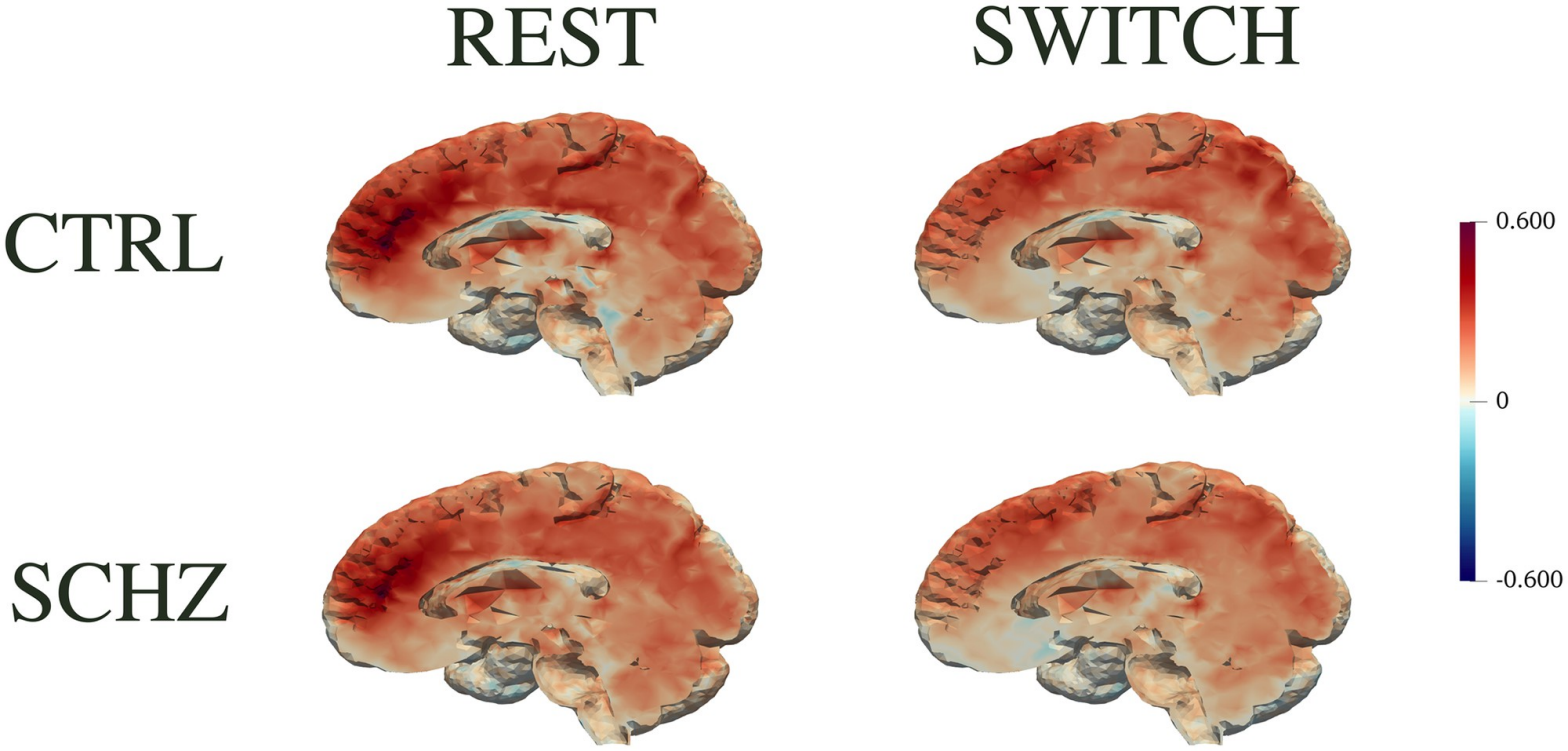
Mean connectivity maps of REST and SWITCH session. The first row depicts the mean z-score of the CTRL group for the two sessions, while the second depicts the mean z-score of the SCHZ group.

Before describing the results of the proposed analysis pipeline, we show that simple depiction of raw connectivity fails to highlight differences between clinically different populations. Fig 1 depicts the mean connectivity maps of the two populations, CTRL and SCHZ, during the REST and the SWITCH task. Indeed, these pictures do not capture any visible difference in the two populations.

For both sessions, we extract four SF-PCs, named SF-PC*j*_REST and SF-PC*j*_SWITCH, for *j* = 1, …, 4, respectively. We obtain a total explained variance of 20.40% for the REST session (cumulative explained variance for each component: 13.49%, 16.16%, 18.88%, 20.40%) and 20.54% for the SWITCH session (cumulative explained variance for each component: 15.24%, 18.83%, 19.38%, 20.54%).

The AUC resulting from the LDA of the scores of the four SF-PCs is 63.5% for REST and of 65.5% for SWITCH. Standard MV-PCA has the same discrimination power, having an AUC of 63.6% for REST and of 65.4% for SWITCH.

More generally, for the two considered sessions, SF-PCA and MV-PCA have the same classification performances also when considering other classification techniques, such as quadratic discriminant analysis, support vector machines and random forests. Random classification of each subject as healthy or affected by schizophrenia leads to an AUC of 54.6% for REST and of 53.6% for SWITCH.

**Fig 2:**
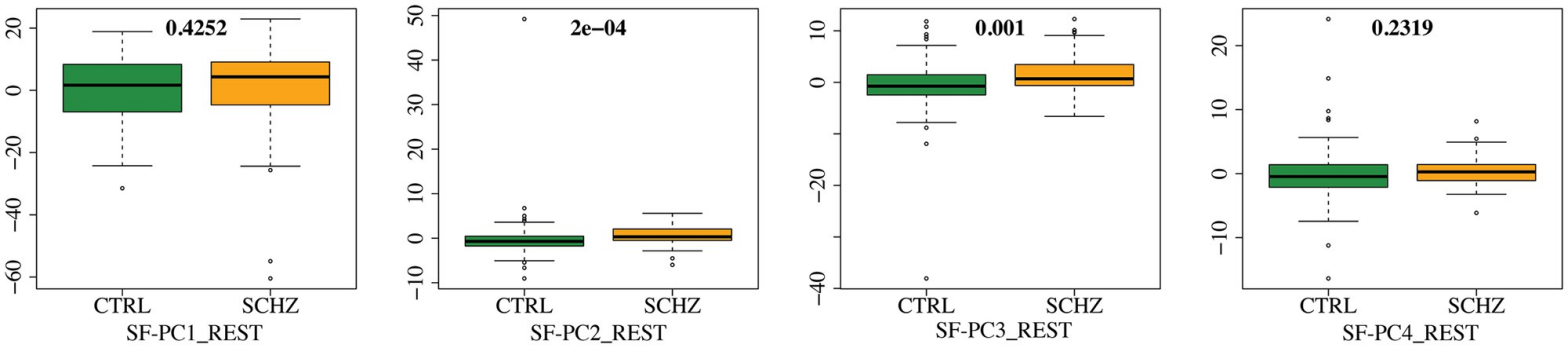
SF-PC scores for REST. Boxplots of scores of the first four SF-PC functions obtained by the proposed SF-PCA, computed on the entire population during REST, and plotted separately for CTRL and SCHZ groups. P-values of Wilcoxon tests that compares the distributions of the PCs scores for CTRL and SCHZ groups.

**Fig 3:**
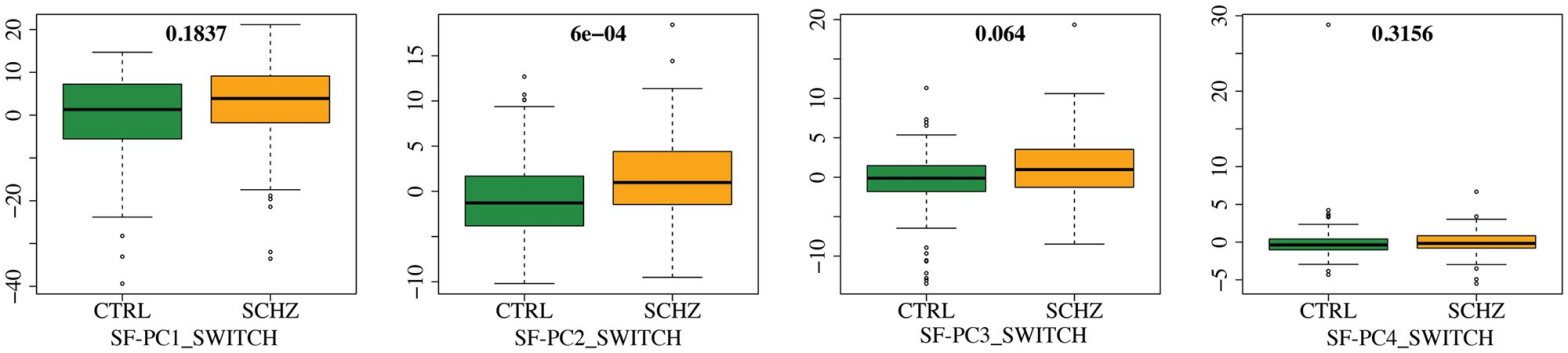
SF-PC scores for SWITCH. Same as Fig 2 but for SWITCH.

To better investigate the distributions of the SF-PC scores in the two population subgroups, in Figs 2 and 3 we display the boxplots of the SF-PCs scores, plotted separately for CTRL and SCHZ, alongside with the results of the correspondent Wilcoxon tests that compare the distributions of the scores for CTRL *vs* SCHZ groups, in REST and SWITCH sessions respectively. SF-PC2_REST, SF-PC3_REST and SF-PC2_SWITCH result significantly different, with p-values respectively: *p* = 2 ⋅ 10^−4^ for SF-PC2_REST, *p* = 1 ⋅ 10^−3^ for SF-PC3_REST, and *p* = 6 ⋅ 10^−4^ for SF-PC2_SWITCH. Similarly, for standard MV-PCA, the scores that are significantly different according to the Wilcoxon tests are: MV-PC3_REST (*p* = 8 ⋅ 10^−4^), MV-PC4_REST (*p* = 2 ⋅ 10^−3^), MV-PC2_SWITCH (*p* = 6 #x22C5; 10^−4^), and MV-PC3_SWITCH (*p* = 2 ⋅ 10^−3^).

**Fig 4:**
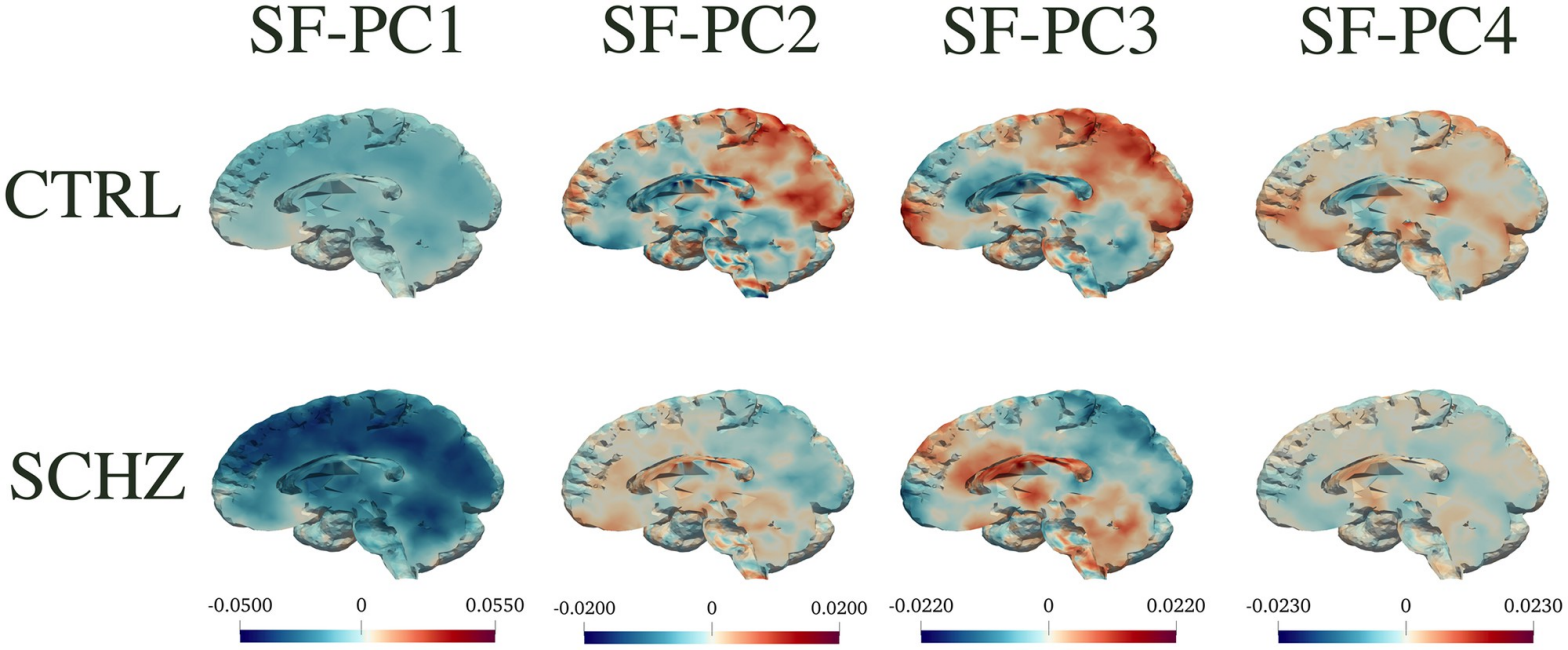
SF-PC functions for REST. Visualizations of the first four SF-PC functions obtained by the proposed SF-PCA, computed on the entire population during REST, and plotted separately for CTRL and SCHZ groups. Top: *median*(*s*_*CTRL*_) ⋅ SF-PC. Bottom: *median*(*s*_*SCHZ*_)⋅ SF-PC. The figure highlights the median behaviour on each component, for subjects in the two populations.

**Fig 5:**
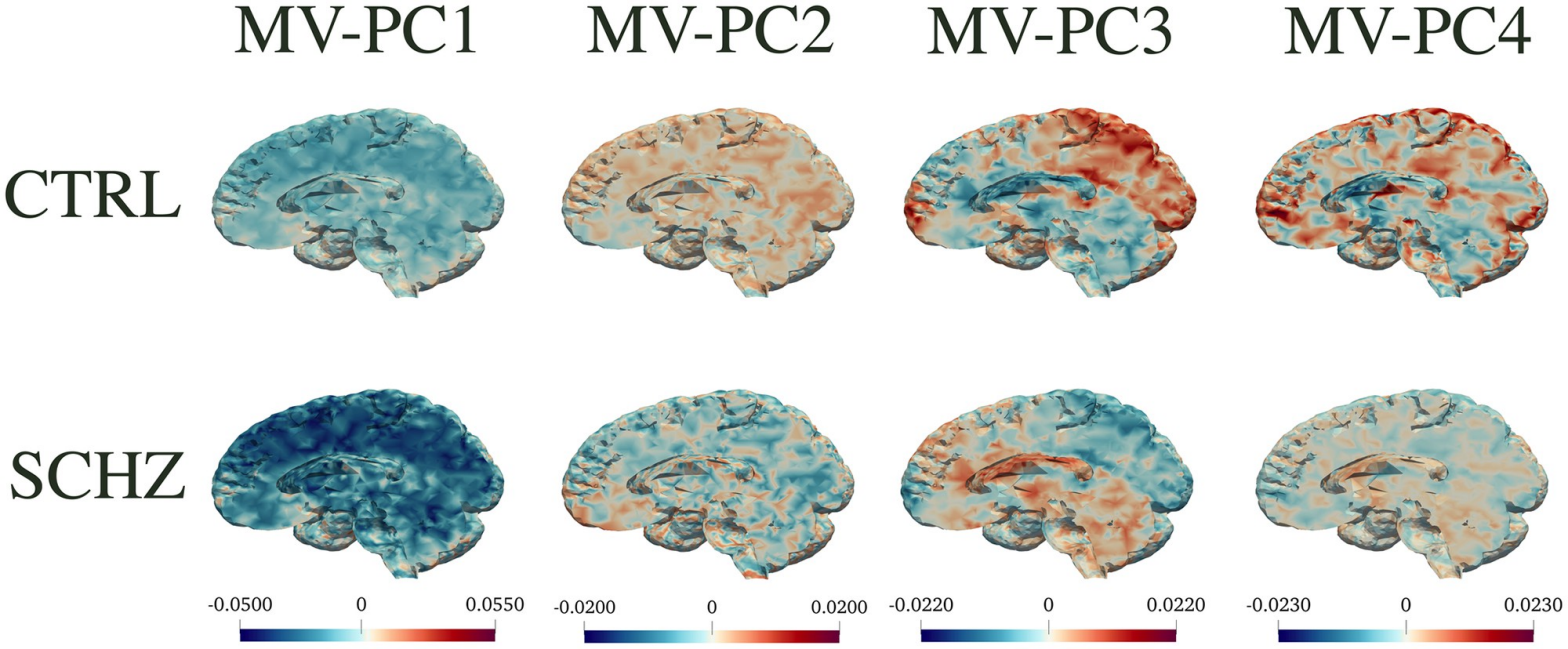
MV-PC for REST. Same as Fig 4 but for standard MV-PCA.

**Fig 6:**
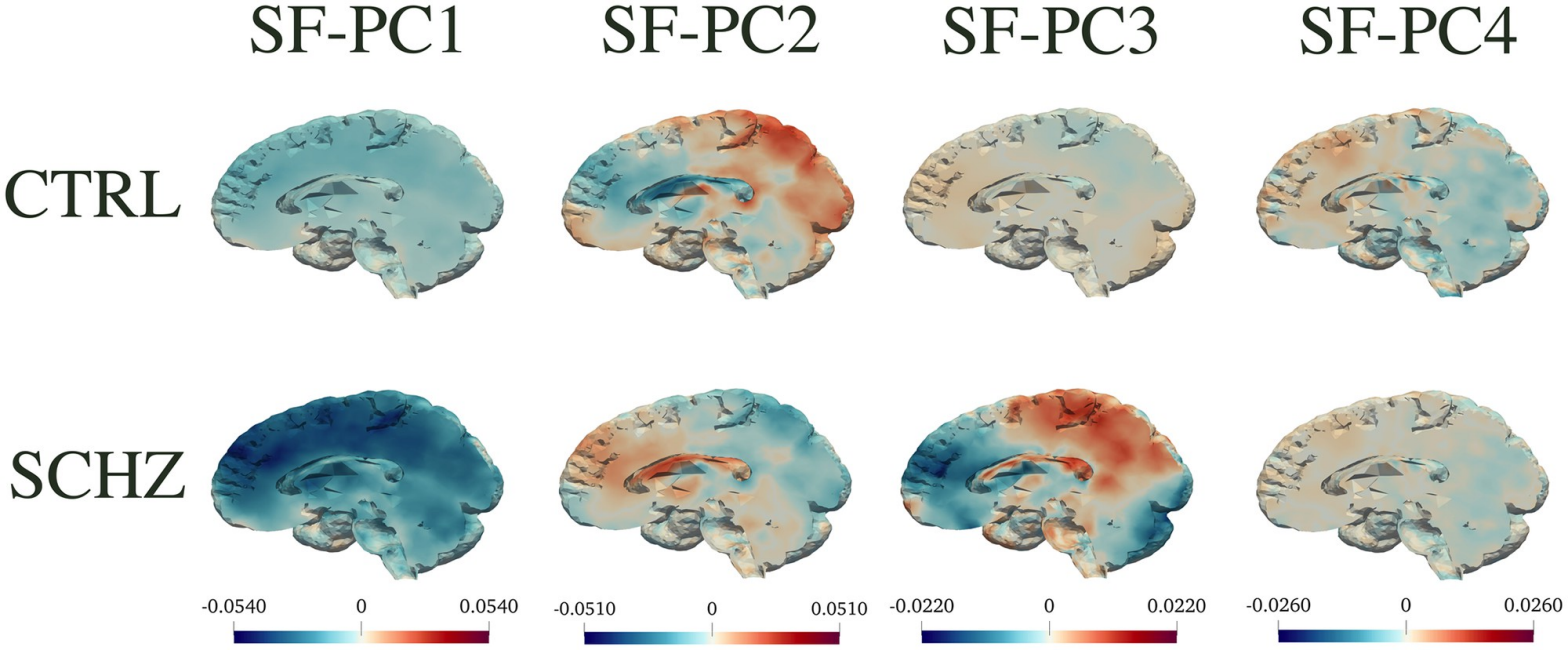
SF-PC functions for SWITCH. Same as Fig 4 but for SWITCH.

To interpret the principal components and visualize their impact on different brain regions, we plot the median behaviour along each component, for subjects in the two populations. In particular, Figs 4 to 6 display in the top panels the *median*(*s*_*CTRL*_) ⋅ *PC*_*j*_ and in the bottom panels the *median*(*s*_*SCHZ*_) ⋅ *PC*_*j*_, where *s* are the scores relative to each principal component. Figs 4 and 5 depict such representations for SF-PCs and MV-PCs for REST; Fig 6 shows the same representation for the SF-PCs for SWITCH (whilst the MV-PCs for SWITCH are not reported for sake of space). These pictures show how each connectivity pattern extracted by the principal component is expressed in the two groups.

We now explore the distributions of the scores for males and females, and across age. For the REST session, the distributions of scores of the 4 computed SF-PCs are not significantly different in males vs females. For the SWITCH session, the distribution of the scores for SF-PC3_SWITCH is different in males vs females, with *p* = 9 ⋅ 10^−3^. For what concerns the Pearson correlation between the PCs scores and the subjects’ age, the maximum correlation present in absolute value is *r* = 0.33.

## Discussion

### Healthy vs affected by schizophrenia: Mean connectivity maps

Fig 1 depicts the mean maps of the two populations. This qualitative representation fails to detail the patterns characterizing different clinical groups. The only noticeable aspect is that the global functional connectivity for the SCHZ is slightly lower during REST as well as during the SWITCH task, coherently with what highlighted in literature (see, e.g., [50, 54]). The compliance with literature suggests that our experimental population, although of limited size, is nevertheless representative of the pathology under study.

### Entire population: Common modes of variation

Through SF-PCA we extract *K* = 4 SF-PCs for each session on the entire population, meaning that each subject is associated with 4 SF-PC_REST scores and 4 SF-PC_SWITCH scores. The percentage of explained variance (that is for both sessions approximately 20%) must be evaluated in the light of the present work’s aim, which is individuating common patterns in connectivity and evaluating possible differences between the two population subgroups, rather than explaining the most part of variability in the dataset. In fact, a dimension reduction from an initial set of approximately 36 000 covariates to 4 would, inevitably, be ineffective, in terms of explained total variance. Even more so when taking into account the extent of interpersonal differences in neural data, and considering the heterogeneity of manifestation and pathology evolution in schizophrenia.

### Healthy vs affected by schizophrenia: Different expressions of the common modes of variation

The proposed SF-PCs has the same discrimination power as standard MV-PCA (about 63% for REST and 65% for SWITCH), but has the valuable advantage of identifying clearly interpretable patterns. In any case, we point out that our objective is not classification, but rather than the identification of clinically relevant patterns in the main modes of variation of functional connectivity. Discrimination on the PC scores indicates these patterns capture significant differences in the two subpopulations, highlighting aspects related to the pathology.

The SF-PCs appear differently expressed in the two subpopulations, as shown by Figs 4 and 6, which plot the median patterns in two groups. At this point, we assess the neurological interpretation of the SF-PCs, with a special emphasis on those diverging between groups. As it is common in classic MV-PCA, the first SF-PCs for REST and SWITCH, i.e., SF-PC1_REST and SF-PC1_SWITCH, depict a grand mean of the entire population. For what concerns the REST, two SF-PCs, SF-PC2_REST and SF-PC3_REST, present different distributions of the scores between CTRL and SCHZ, with p-values *p* = 2 ⋅ 10^−4^ and *p* = 1 ⋅ 10^−3^, respectively; see Fig 2. SF-PC2_REST shows a distinct pattern associated with well defined regions; see Fig 4. In particular, it involves, on the one hand, the superior parietal gyrus, and, on the other, the cuneus and the medial prefrontal cortex. The first region is part of the Executive Control Network, while the latter has been associated with the Default Mode Network. SF-PC2_REST may thus suggest a difference in the correlation and uncoupling of such networks among the groups, a phenomenon known to affect schizophrenia patients (see, e.g., [55]). SF-PC3_REST captures additional contrasts among the areas highlighted by SF-PC2_REST, substantiating the importance of the modifications affecting the Default Mode Network in schizophrenia patients. Moreover, a hub of activation in the prefrontal region is highlighted for the SCHZ group. This brain region plays a pivotal role in the cognitive impairment, and in other manifestations of this pathology [56]. As a comparison, we note that standard MV-PCA, although reaching the same classification performances as SF-PCA, it fails to capture clearly identifiable patterns. For instance, while the involvement of the Default Mode Network is partially captured by MV-PC3_REST, no relevant pattern is highlighted by MV-PC2_REST; see Fig 5. Overall, all the extracted MV-PCs are highly noisy, making it difficult to propose interpretations.

For what concerns the modes of variations associated with the SWITCH paradigm, the scores of SF-PC2_SWITCH point out a difference in the SF-PC’s expression, with a p-value *p* = 6 ⋅ 10^−4^; see Fig 3. The pattern identified by SF-PC2_SWITCH, depicted in Fig 6, mainly involves the frontal and parietal regions, coherently with previous findings regarding task-switching paradigm (see, e.g., [57]), showing how the mechanisms and connections involved in this activity are impaired by the pathology under study.

It thus emerges that the modes of variations computed through SF-PCA are informative of the pathology in analysis, as they successfully highlight relevant differences between the populations, as also confirmed by the compliance with previous literature.

### Gender and age: No significant difference in the expressions of the common modes of variation

It is very interesting to note that the scores of the obtained PCs are not significantly different between males and females (*p* > 0.05), with the exception of SF-PC3_SWITCH (*p* = 9 ⋅ 10^−3^), and are also unrelated to age (max Pearson’s correlation in absolute value *r* = 0.33). This supports that the signal captured by SF-PCA is related to the pathology under study, rather than these other covariates. Hence, the unbalance in the considerate population does not hinder the validity of the obtained results. SF-PC3_SWITCH indicates a hub of activation in the prefrontal region, which might be an interesting phenomenon to explore in further studies.

## Conclusions

In the present work, we proposed to extend the use of SF-PCA method, previously validated for FC maps computed over the cortical surface, for the analysis of grey matter volume data. The FC maps considered refer to two separate fMRI sessions: one resting-state and one during a task based on the switching paradigm.

The adequacy of the proposed approach is assessed through classification performance and compliance of the extracted features with previous findings. During both sessions, the extracted SF-PCs scores significantly differ in the two subpopulations of CTRL and SCHZ. Linear Discriminant Analysis of the SF-PC scores discriminates the subjects’ diagnosis with an above-chance Area Under the Curve. More significantly, the extracted features are coherent with previous literature. In particular, during REST, the SF-PCs highlight the disruption of the physiological alternation between the Default Mode and the Executive Control networks, one of the key mechanisms in schizophrenia’s physiopathology. Analogously, SF-PC2_SWITCH identifies the regions known to be involved in task-switching and impaired by schizophrenia. Unlike the standard MV-PCA, the proposed SF-PCA, succeeds in identifying readable and relevant patterns. We believe that the gained interpretability is a valuable asset for studies in the field.

Given the unbalance of the experimental population, in terms of sex and age, we have verified that the main modes of variations do not significantly differ across age and gender. The only resulting difference between genders, indicated by SF-PC3_SWITCH, does not overlap with phenomena related to the pathology; hence, the population unbalance does not invalidate our results. In clinical applications, it would nevertheless be desirable to have more balanced groups. Moreover, an improvement in the sample size and in data quality and resolution would certainly lead to the individuation of additional patterns and to higher explained variability and discrimination performances. In any case, we stress that the aim of the present work is not to derive clinical evidence about schizophrenia, but rather to propose an innovative data analysis method that can be profitably applied in neuroscience research. In particular, we have shown that SF-PCA is a valuable tool to explore the variability in functional connectivity maps in the complex tridimensional domain of the grey matter. Furthermore, by reducing the dimensionality of data and simultaneously maintaining their interpretability, SF-PCA also addresses some of the computational issues, very relevant in the field. For these reasons, SF-PCA may be especially useful for the study of brain connectivity in connection with neurological and psychiatric illnesses, as it may identify significant differences between subpopulations, and shed new light on the considered pathology. Moreover, the ability of the proposed method to include in the data analysis the volume brain anatomy constitutes a relevant addition with respect to the available literature. In future research, we aim to extend this valuable modeling feature to other data analysis techniques, commonly used in neuroimaging studies, such as independent component analysis and generalized linear models, and develop their anatomically compliant versions. Furthermore, following the approaches described in [58, 59] for cortical surface data, we may aim at developing methods capable to handle subject-specific anatomies. This would enable to jointly analyse functional aspects and subject-specific structural changes induced by the considered pathology [60], such as atrophy.

Finally, the present approach could be extended to different ways of computation of FC maps, for instance through nonlinear metrics, or by considering activation maps. In future works, we plan to apply the hereby validated method to further pathologies and to employ the extracted SF-PCs to evaluate the correlation between connectivity and the outcomes of clinical assessment tests. We also aim to approach only pathological samples, to be able to explore in more detail the heterogeneity characterizing psychiatric illnesses. Finally, we plan to assess the proposed approach in clinical settings, to monitor patients enrolled in rehabilitation programs.
